# Color-Shape Associations Revealed with Implicit Association Tests

**DOI:** 10.1371/journal.pone.0116954

**Published:** 2015-01-27

**Authors:** Na Chen, Kanji Tanaka, Katsumi Watanabe

**Affiliations:** Research Center for Advanced Science and Technology, The University of Tokyo, Tokyo, Japan; University of Bath, UNITED KINGDOM

## Abstract

Kandinsky proposed a correspondence theory that suggests associations between specific colors and shapes (i.e., circle-blue, square-red, triangle-yellow). Makin and Wuerger tested the theory using the Implicit Association Test (IAT) and did not find clear evidence for Kandinsky’s color-shape associations among British participants. In the present study, we first replicated the previous study among Japanese participants and found similar results to those of Makin and Wuerger, showing little support for Kandinsky’s theory. In the subsequent experiment, we tested another set of color-shape associations that had been revealed by using an explicit matching method (circle-red, square-blue, triangle-yellow) in Japanese participants. The IAT tests showed that response times were significantly faster when circle-red, square-blue, and triangle-yellow combinations were mapped onto the same response key, rather than different key combinations, indicating that these color-shape combinations were encoded. These results provide the first empirical evidence that color-shape associations can be measured by indirect behavioral methods, and in particular, Japanese people’s color-shape associations (circle-red, square-blue, triangle-yellow) can be observed by both direct and indirect experimental methods.

## Introduction

Kandinsky, a renowned abstract painter, proposed a correspondence theory suggesting an association between primary colors and shapes, according to which a circle is matched with the color blue, a square is matched with the color red, and a triangle is matched with the color yellow. He argued that the correspondence between colors and shapes was mediated by an inherent relationship between the colors and angles of the shapes [1–2]. However, the correspondence theory has evoked opposition since its proposal. Indeed, several studies have failed to replicate Kandinsky’s correspondence theory [3–8]. For example, Jacobsen used a modified Kandinsky questionnaire and found color-shape combinations of circle-yellow, square-blue, and triangle-red in German samples, suggesting that color-shape associations were based on everyday knowledge and experience [3–4]. Albertazzi et al. used a matching task to investigate color-shape associations in Italian participants, and found that both circle and square were matched with red, while a triangle was matched with yellow. They argued that color-shape associations might be determined by semantic information such as the “warmth” and “lightness” [5]. Chen et al. reported that Japanese participants showed color-shape associations similar to those of Italian participants (Japanese color-shape association: circle-red, square-blue, and triangle-yellow) [6], suggesting that color-shape associations might stem from shared semantic information between colors and shapes (e.g., warm/cold) as Albertazzi et al. These studies indicate that there exist non-random associations between colors and shapes, and these color-shape associations might be influenced by the semantic information associated with visual features and people’s learning experience.

In addition to the direct matching tasks in the aforementioned studies, some studies have adopted indirect behavioral measures to investigate color-shape associations. Moreover, studies using indirect behavioral methods have also failed to find clear evidence for Kandinsky’s color-shape associations [9–11]. Kharkhurin revisited Kandinsky’s color-shape associations in two experiments: one used with a priming technique and the other used a recognition test. He first tested whether an earlier-presented color stimulus influenced the discrimination of a later-presented shape stimulus and hypothesized that the response time (RT) would be faster in congruent conditions in which the priming color was associated with the shape. Then, in the second experiment, he measured RTs in a discrimination task using a shape filled with various colors and hypothesized that discrimination times would be faster for shapes filled with the associated color. However, both experiments failed to reveal any acceleration of RTs in the congruent conditions in Kandinsky’s correspondence theory [9]. Makin and Wuerger used implicit association tests (IAT) to examine Kandinsky’s correspondence theory. They did not find clear evidence of correspondence, but found marginal effects in only one combination (square-red and triangle-yellow associations) among the three combinations they tested (the other two combinations: circle-blue and square-red, circle-blue and triangle-yellow associations) [11].

Taken together, studies have shown little support for Kandinsky’s color-shape associations using either direct matching or indirect behavioral experimental methods [3–11]. One possible reason might be that Kandinsky’s correspondence theory is not universal or simply incorrect. Previous studies using explicit matching task have shown that there exist other color-shape associations [5–6]. Then, the question is: Is it possible to demonstrate color-shape associations in the indirect behavioral measures? One possibility is that these associations are constructed only through subjective introspections, and may not be measured through indirect behavioral performance reflecting quick judgments. But, the other possibility that the indirect behavioral measures have not been tested for color-shape associations with empirical supports remains [6, 11].

The present study aimed to examine whether two forms of color-shape associations reported in direct matching tasks in the previous studies could be observed by an indirect behavioral method: one referred Kandinsky’s correspondence theory [11] and the other referred to Japanese color-shape association [6]. For this, we adopted the IAT, which is known to be a useful tool to reveal people’s unconscious associations between dimensions by response time [12–13]. In the IAT experiment, participants have to classify four stimulus categories by pressing two different keys. Participants are instructed to press one key rapidly and accurately for one pair of visual stimuli and to press the other key for the other pair of visual stimuli. If the two stimuli assigned to the same key are associated (congruent condition), the RT should theoretically be shorter than in the condition in which two stimuli assigned to the same key are unassociated (incongruent conditions). Hence, the IAT reveals associations at the level of response selection. It has been widely used to assess the strength of associations between different dimensions [14–16]. In the present study, we used multiple IAT tasks to explore the associations between colors and shapes [11, 16]. We first replicated the study of Makin and Wuerger [11] to examine whether Kandinsky’s correspondence theory could be observed in Japanese participants. Having negative results in the first experiment, in the second experiment, we explored a different pattern of color-shape associations, reflecting our previous findings of color-shape associations among Japanese participants [6].

## Experiment 1

### Method

#### Participants

Thirty-eight Japanese college students took part (24 males, mean age = 21.5, SD = 2.1). All participants had normal or corrected-to-normal visual acuity and normal motor functions, and were naïve to the purpose of this study. This experiment, as well as the subsequent experiments, were approved by the institutional review board (IRB) of The University of Tokyo, and conducted in accordance with the ethical standards in the 1964 Declaration of Helsinki. Written informed consent was obtained from all participants in advance.

#### Apparatus and stimuli

**Figure 1:**
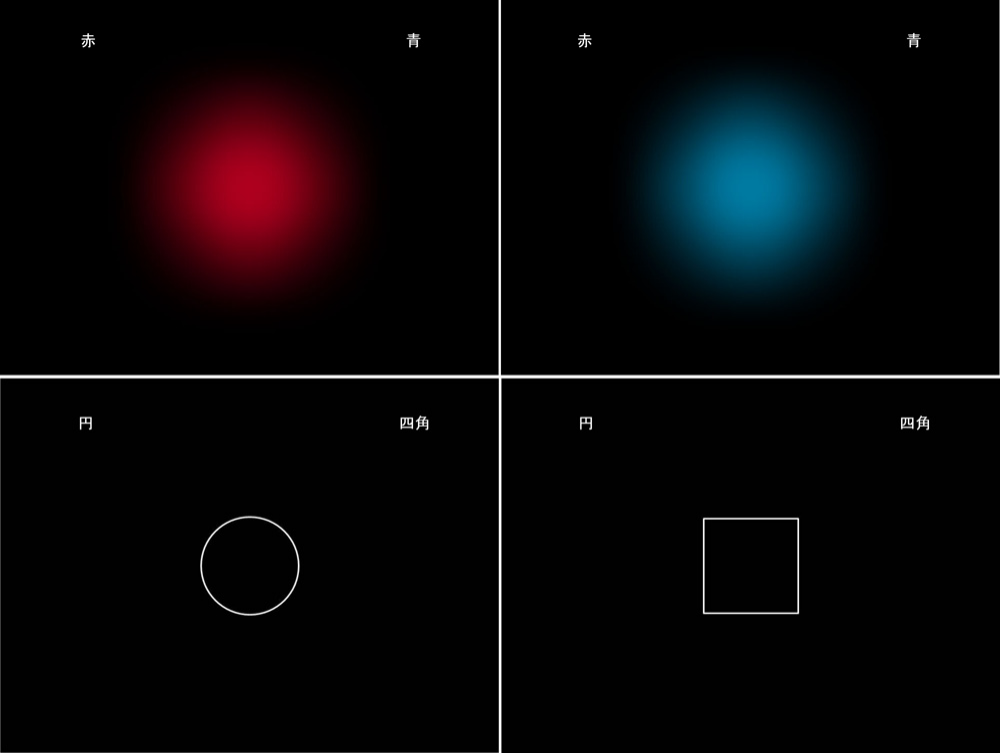
Example stimuli used in the experiment. The words of shapes or colors appeared on the left and right sides of the screen. For example, “赤” (red), “青” (blue), “円” (circle), and “四角” (square) were used as cue words to remind participants of the key press.

Stimuli were presented at 60cm on a 1280 × 1024 pixel CRT monitor, with a refresh rate of 60 Hz. E-Prime 2.0 software (Psychology Software Tools, Inc.) was used to present the stimuli and collect the data. The three shape stimuli were all white line drawings (with a width of 26mm, 0.03° visual angle) on a black background (8 cd/m^2^). The circle was 4.8° in diameter, the square was 4.8° (in height) × 4.8° (in width), and the triangle was 4.8° (in height) × 5.8° (in width). The three shape stimuli were always presented in the upward orientation. Color patches were ~9.5° wide (Gaussian-modulated). The colored Gaussian patches were measured using PR-655 (Photo Research, Chatsworth, CA, USA) and each color was consecutively measured 10 times and averaged. The color information was as follows: yellow: L* = 46.44, a* = -2.57, b* = 53.73; red: L* = 20.99, a* = 37.82, b* = 27.66; blue: L* = 27.79, a* = -14.58, b* = -15.12. Example stimuli are shown in Fig. 1.

#### Procedure

The experiment was carried out in a laboratory with dimmed lighting conditions (1 lux on the wall). The structure of the IAT was based on the study conducted by Makin and Wuerger [11]. The experiment was composed of three IAT tasks, each consisting of 8 blocks (Tables 1 and 2). During the task, participants were asked to discriminate visual stimulus of color or shape by pressing two keys (i.e., left key “z” and right key “m”) in the keyboard using the two index fingers. In the first block (Block 1), participants were trained to discriminate a shape stimulus by pressing a key (for example, the left key for circle and the right key for square in IAT 1; Table 1). In the second block (Block 2), participants were trained to discriminate a color stimulus (e.g., the left key for blue, the right key for red) by pressing a key. In the third and fourth blocks (Blocks 3 and 4), color and shape discriminations were combined, and responses were mapped according to Kandinsky’s color-shape associations (e.g., the left key for circle or blue, the right key for square or red; congruent blocks). In the following two training blocks (Blocks 5 and 6), participants were instructed to learn a shape-key mapping opposite to that of the previous blocks (e.g., left key for square, right key for circle). In the last two blocks (Blocks 7 and 8), the response mapping was the opposite of Kandinsky’s color-shape associations (e.g., the left key for square or blue, the right key for circle or red; incongruent blocks).

**Table 1:** Sequence of blocks and response mappings (example from IAT 1).

**Block**	**Trials**	**Condition**	**Left key response**	**Right key response**
1	20	Training 1	Circle	Square
2	20	Training 2	Blue	Red
3	20	Congruent 1	Circle or blue	Square or red
4	40	Congruent 2	Circle or blue	Square or red
5	20	Training 3	Square	Circle
6	20	Training 4	Square	Circle
7	20	Incongruent 1	Square or blue	Circle or red
8	40	Incongruent 2	Square or blue	Circle or red

**Table 2:** The response mappings in the IATs of Experiments 1 and 2.

	**IAT 1**		**IAT 2**		**IAT 3**	
**Blocks**	**Left key**	**Right key**	**Left key**	**Right key**	**Left key**	**Right key**
Congruent blocks in Exp. 1	Circle	Square	Circle	Triangle	Square	Triangle
	Blue	Red	Blue	Yellow	Red	Yellow
Incongruent blocks in Exp. 1	Square	Circle	Triangle	Circle	Triangle	Square
	Blue	Red	Blue	Yellow	Red	Yellow
Congruent blocks in Exp. 2	Circle	Square	Circle	Triangle	Square	Triangle
	Red	Blue	Red	Yellow	Blue	Yellow
Incongruent blocks in Exp. 2	Square	Circle	Triangle	Circle	Triangle	Square
	Red	Blue	Red	Yellow	Blue	Yellow

In each block, the stimuli were presented in a novel random order for each participant. Cue words shown on the top two sides of the screen were used to remind participants which side of key should be pressed to report their answers. For example, in the first training block of IAT 1, the words “円” (circle) and “四角” (square) appeared on the top left and top right sides of the screen according to the response mapping. When participants pressed the wrong button, the word “Error” appeared centrally in red, and a beep sounded at the same time. Before each block, participants were appropriately informed of the task, stimuli, response mapping, and cue words. For instance, the instruction for block 1 in IAT 1 was as follows. “You are going to take part in a test measures your response time in discriminating visual stimulus for colors and shapes. In this block, when you see a circle (○), please press key “z” with your left index finger. when you see a square (□), please press key “m” with your right index finger. The cue words of “円” (circle) and “四角” (square) will be appeared at the top left or right sides of the screen to remind you which side of key to press. Please press the key as accurately and fast as possible. When you press the wrong key, a red word “ERROR” will appear and a beep will sound.” For another example as in the block 3, the main instruction would be changed into “when you see a circle (○) or color blue, please press key “z” with your left index finger; when you see a square (□) or color red, please press key “m” with your right index finger. The cue words of “円/青” (circle/blue) and “四角/赤” (square/red) will be appeared at the top left or right side of the screen to remind you which side of key to press.”

The order of the congruent and incongruent blocks was counterbalanced between participants; half of the participants were presented the congruent blocks first, and the other half of participants were presented the incongruent blocks first [11]. Each participant completed three types of IAT tasks (response mappings are shown in Table 2). The order of the three IAT tasks was also counterbalanced across participants. Each IAT task took about 7 minutes, and participants rested 3 minutes between tasks. The RTs and the number of error trials were collected for data analysis. The whole experiment took about 30 minutes.

At the end of the experiment, participants were instructed to complete a paper questionnaire. On the questionnaire, three shapes (i.e., circle, square, and triangle) and three colors (i.e., red, blue, and yellow) were presented in two rows, with the locations counterbalanced across participants. Then, participants were asked to intuitively connect the matched colors and shapes by drawing three lines, resulting in no overlapped colors or shapes and six types of color-shape associations (e.g., circle-red, square-blue, triangle-yellow; Table 3).

**Table 3:** Explicit questionnaire results in Experiment 1 and Experiment 2.

**Label**	**Circle**	**Square**	**Triangle**	**Exp. 1 (N = 38)**	**Exp. 2 (N = 24)**
1	Red	Blue	Yellow	14 (36.84%)	13 (54.16%)
2	Blue	Red	Yellow	14 (36.84%)	3 (12.5%)
3	Red	Yellow	Blue	3 (7.89%)	4 (16.67%)
4	Blue	Yellow	Red	2 (5.26%)	3 (12.5%)
5	Yellow	Blue	Red	2 (5.26%)	0 (0%)
6	Yellow	Red	Blue	3 (7.89%)	1 (4.17%)

#### Analysis

We adopted the D score measure, which was widely used in the IAT studies [11, 12, 13, 16, 17]. We computed the D score of every IAT task for each participant, with reference to the study of Makin and Wuerger [11]. The step-by-step procedure for computing D scores was as follows:

1) Include trials from block 3, 4, 7, 8 in analysis.
2) Compute mean RT and standard deviation for each individual in each IAT and each congruent and incongruent condition separately.
3) Compute boundary values by adding or subtracting 2 *SD*s to the mean RT.
4) Include the trials [mean − 2 *SD*, mean + 2 *SD*].
5) Compute the mean RTs from block 3, 4, 7, 8.
6) Compute one pooled *SD* for trials in blocks 3 and 7 as *SD*_(3,7)_, another one for blocks 4 and 8 as *SD*_(4,8)_.
7) Compute two difference RTs in block 3 and 7 (RT 7 − RT 3) and block 4 and 8 (RT 8 − RT 4).
8) Divide each difference of RTs by its associated pooled *SD* from step 6, producing two D scores (e.g., D score_(3,7)_ = (RT 7 − RT 3) / *SD*_(3,7)_).
9) Compute the average of two D scores (i.e., D score_(3,7)_ and D score_(4,8)_ in step 8), generate a final D score for each participant in each IAT.

This procedure was repeated for the three IAT tasks; therefore, each participant had three D scores for the three IATs. The D score represents the difference between the means of congruent and incongruent blocks, as a function of the standard deviation of the distribution. For participants who did congruent first, a positive D score value indicates support for the proposed hypothesis, and a negative value indicates that the participant showed the opposite associations to the hypothesis.

### Results and Discussion

We excluded error trials (3.6%) and trials with RTs longer than 2000 ms (0.8%) from data analysis. We calculated D-scores and response times in congruent and incongruent blocks from each of the three IAT tasks. All individual-level data including participant information, D score, RT and error rate are provided in S1 Table.

#### D scores

**Figure 2:**
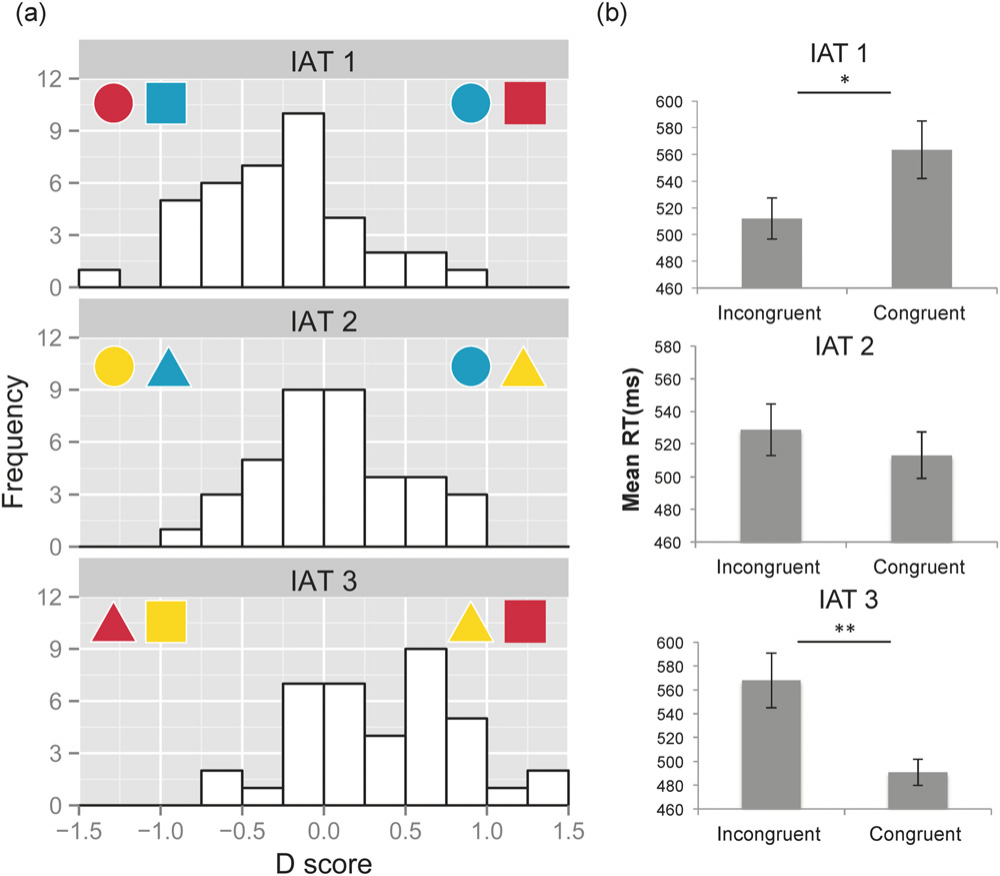
Results of D scores and mean RTs in Experiment 1. **(a) Distribution of D scores for the three IAT tasks with Kandinsky’s color-shape associations.** Positive D scores indicate that trials in the left color-shape combinations were faster than those in the right. **(b) Mean RTs in congruent and incongruent blocks for the three IAT tasks.** Error bars represent the standard errors of the mean.

One-sample *t*-tests with Bonferroni corrections (as there were six tests, the adjusted *p* value was derived from *p* value of each *t*-test multiplied by six, and then compared with the significant level of 0.05) were performed to compare D scores to zero (Fig. 2a). In IAT 1, D scores were distributed in the left (i.e., unexpected) direction (IAT 1: *t*[37] = 3.42, adjusted *p* < .01; mean D score = -0.26). This result indicates an association pattern opposite to that of Kandinsky’s color-shape associations; our participants associated circle with red and square with blue. In IAT 2, D scores were distributed around zero (IAT 2: *t*[37] = 0.88, adjusted *p* > .99; mean D score = 0.06). Thus, participants did not show any specific associations in this type of color-shape combination (i.e., circle/triangle and blue/yellow). In IAT 3, D scores were distributed in the right (i.e., expected) direction (IAT 3: *t*[37] = 4.65, adjusted *p* < .01; mean D score = 0.38), which suggested a congruency effect of triangle-yellow and square-red associations (i.e., consistent with Kandinsky’s color-shape associations).

#### Response time

We calculated and compared mean RTs in congruent and incongruent conditions (Fig. 2b). Paired *t*-tests with Bonferroni corrections showed similar patterns in the results of D scores. In IAT 1, participants responded faster in the incongruent (512 ms) than in the congruent (563 ms) conditions (*t*[37] = 3.04, adjusted *p* <.05). In IAT 2, there was no significant difference between the congruent (513 ms) and incongruent (529 ms) conditions (*t*[37] = 1.46, adjusted *p* = .92). In IAT 3, participants responded faster in the congruent (491 ms) than in the incongruent (568 ms) conditions (*t*[37] = 4.02, adjusted *p* <.01).

#### Comparison with Makin and Wuerger [11]

Here, we discuss differences of the present results and Makin and Wuerger who run the same experiments with a different set of participants. In the present study, we recruited thirty-eight Japanese college students (24 males, mean age = 21.5, *SD* = 1.5) and in Makin and Wuerger, they recruited thirty-six British participants (age 18–45, 11 males) who were mostly undergraduate or postgraduate students at the university of Liverpool [11].

In the present IAT 1, the D scores were significantly shifted to the left direction (mean D score = -0.26), and the RTs in the incongruent blocks (mean RT: 512 ms) were significantly faster than those in the congruent blocks (563 ms). This indicates that most of participants associated circle with red and square with blue (i.e., opposite of Kandinsky’s color-shape associations). Makin and Wuerger [11] used the same set of color and shape stimuli (circle/square and red/blue in their IAT 2), and D scores in their study were distributed around zero (mean D score = -0.04), and there was no significant difference in RTs (*t*[35] = -1.502, *p* = 0.142, congruent = 630ms vs. incongruent = 601 ms). This discrepancy may need further investigations. Nevertheless, these results indicate that most people do not have circle-blue and square-red associations proposed by Kandinsky. In addition, the previous studies using direct matching task reported circle-red and square-blue associations, which was opposite to Kandinsky’s association [6, 7, 9].

In IAT 2, the D scores were distributed around zero (mean D score = 0.06) and RTs did not show any differences between the congruent (513 ms) and incongruent (529 ms) conditions. It is likely similar to the null result in Makin and Wuerger [11] (mean D score = -0.08; RT: 609 ms vs. 588 ms in the congruent and incongruent conditions, respectively, in their IAT 1). These results indicate that there was little congruency effect consistent with Kandinsky’s circle-blue and triangle-yellow associations.

In IAT 3, we observed that D scores were significantly shifted to the positive direction (mean D score = 0.38), and participants responded faster in the congruent (491 ms) than in the incongruent (568 ms) condition, suggesting a congruency effect of square-red and triangle-yellow associations. This result supports Kandinsky’s correspondence theory. Makin and Wuerger also observed a marginally significant difference in their IAT 3 for this color-shape combination; D scores were marginally shifted to the right (*p* = 0.051, mean D score = 0.13) and RTs in the congruent conditions (575 ms) were faster than in the incongruent conditions (621 ms) [11].

#### Explicit questionnaire

The results of the explicit questionnaire were shown in Table 3. A chi-square goodness of fit test showed that our participants made some color-shape combinations more frequently than others (*x* ^2^ = 27.99, *df* = 5, *p* <.01). We can see that 14 out of 38 participants (36.84%) made color-shape associations consistent with Kandinsky’s theory (Label 2 of Table 3) and the same number of participants (14 out of 38 participants; 36.84%) chosen Japanese color-shape associations (Label 1 of Table 3). However, the participants who expressed color-shape associations consistent with Kandinsky’s theory may be influenced by the IAT task they had performed [3, 16]. In the present study, we run the IAT first and then the explicit questionnaire because our aim was to investigate whether the proposed color-shape associations could be observed by the IAT, resulting in that the explicit questionnaire was influenced by the priming effect of the IAT paradigm. However, if we run the explicit questionnaire first, and the IAT next, the performance in the IAT would be inevitably affected by the choices in the explicit questionnaire. As the main purpose of the present study was to examine whether color-shape associations could be measured by the IAT, and the explicit questionnaire was used to test whether Japanese color-shape associations [6] could be observed even under the priming effect of the IAT. Therefore, we run the experiments in the present order. In the present experimental paradigms, the congruent color-shape associations appeared twice while the incongruent color-shape associations appeared only once (e.g., red-square appeared in IAT 1 and 3 while blue-square and yellow-square appeared once in IAT 1 and 3, respectively); therefore, the difference of the number of responses might affect the answers of explicit questionnaire to some extent. Hence, here, we mainly discuss the results of participants who showed unmatched color-shape associations with Kandinsky’s theory in the explicit questionnaire (14 participants in the matched group and 24 participants in the unmatched group).

For participants who showed matched color-shape associations, one sample *t*-tests with Bonferroni correction revealed significant differences between the D scores and zero only in IAT 3 (IAT 1: *t*[13] = 2.07, adjusted *p* = .12, mean D score = -0.27; IAT 2: *t*[13] = 1.66, adjusted *p* = .32, mean D score = 0.17; IAT 3: *t*[13] = 3.58, adjusted *p* <.05, mean D score = 0.42). This indicates that participants who endorsed Kandinsky’s color-shape associations in the explicit matching task did not clearly show faster responses in the expected congruent conditions in the IAT. However, one possibility should be considered that some participants chose Kandinsky’s color-shape associations due to the imbalance of the responses while they might have other associations in fact. For this understanding, improvement of experimental paradigms is required in the future study.

For participants who showed unmatched color-shape associations, D scores revealed significant congruency effects in IAT 1 and IAT 3 (IAT 1: *t*[23] = 2.65, adjusted *p* <.05, mean D score = -0.26; IAT 2: *t*[23] = 0.01, adjusted *p* >.99, mean D score = -0.001; IAT 3: *t*[23] = 3.17, adjusted *p* <.05, mean D score = 0.34). The results of IAT 1 showed circle-red and square-blue associations and those of IAT 3 showed triangle-yellow and square-red. These faster responses might be caused by Japanese color-shape associations (circle-red, square-blue, and triangle-yellow; Label 1 in Table 3) because 14 out of 24 participants in the unmatched group (58.33%) chose the association.

#### Interim summary

Overall, in Experiment 1, we replicated the study of Makin and Wuerger [11] in Japanese participants, which suggested little support for Kandinsky’s color-shape associations. In IAT 1, we observed circle-red and square-blue congruent association effects, and in IAT 3, we observed triangle-yellow and square-red congruent association effects. Interestingly, these color-shape associations have been observed in the previous studies using explicit matching tasks [5–6]. Albertazzi et al. asked Italian participants to choose the color that best matched a shape from a color wheel (40 hue colors from Natural Color System), and found that circle and square were associated with red, and a triangle was matched with yellow [5]. Chen et al. replicated the study in Japanese participants and found that circle was associated with red, square was associated with blue, and triangle was associated with yellow [6]. Indeed, in the explicit questionnaire, the circle-red, square-blue, triangle-yellow associations were prominent.

## Experiment 2

In the previous study [6], Chen et al. used an explicit matching task and asked participants to choose a best matched hue color for a geometric shape from 40 color circles. The results showed that participants systematically associated shapes with colors. Chen et al. found that about 34% of participants matched circle with red colors, 33% of participants matched triangle with yellow colors, and 19% of participants matched square with blue colors. Therefore, we reason that the Japanese color-shape associations might be represented as circle-red, triangle-yellow, and square-blue. In Experiment 2, in order to examine whether there were any color-shape associations that could be consistently shown using the IAT, we examined the recently proposed color-shape associations found in Japanese participants (circle-red, square-blue, triangle-yellow; [6]), instead of Kandinsky’s color-shape associations.

### Method

#### Participants

Twenty-four new participants took part in the study (16 males, Mean = 21.0, SD = 1.4). All participants had normal or corrected-to-normal visual acuity and normal color vision.

#### Apparatus and procedure

The apparatus, shape stimuli, and color stimuli were the same with those in Experiment 1. In each IAT task, the congruent combinations of color-shape associations were changed according to the color-shape associations observed in Japanese participants (i.e., circle-red, square-blue, triangle-yellow; see Table 2; [6]). The experimental procedure was also identical to that of Experiment 1.

### Results and Discussion

We excluded error trials (3.9%) and trials with RTs longer than 2000 ms (1.3%) from data analysis. We calculated D-scores and response times in congruent and incongruent blocks from each of the three IAT tasks as in experiment 1. All individual-level data including participant information, D score, RT, and error rate are provided in S2 Table.

#### D scores

**Figure 3:**
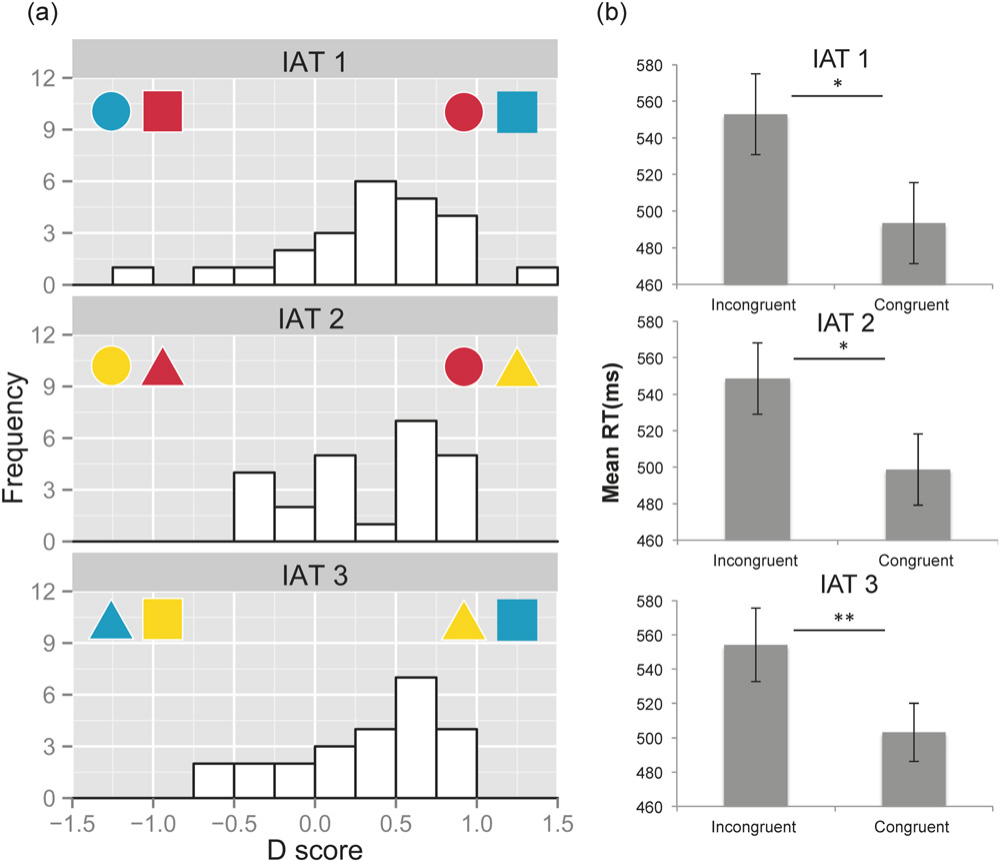
Results of D scores and mean RTs in Experiment 2. **(a) Distribution of D scores for the three IAT tasks with Japanese color-shape associations.** Positive D scores indicate that trials in the left color-shape combinations were faster than those in the right. **(b) Mean RTs in congruent and incongruent blocks for the three IAT tasks.** Error bars represent the standard errors of the mean.

One sample *t*-tests with Bonferroni corrections showed that the D scores of the three IAT tasks were significantly distributed in the right (i.e., expected) direction (IAT 1, *t*[23] = 3.34, adjusted *p* <.05; IAT 2, *t*[23] = 3.27, adjusted *p* <.05; IAT 3, *t*[23] = 3.58, adjusted *p* <.01). Thus, the congruency effect of the color-shape associations modulated RTs (Fig. 3a). This indicates that the participants associated circle with red, square with blue, and triangle with yellow, which was consistent with the color-shape associations observed in Japanese samples.

#### Response time

We calculated mean RTs for the congruent and incongruent blocks (Fig. 3b). Paired sample *t*-tests with Bonferroni corrections showed that RTs were significantly faster in the congruent than in the incongruent conditions in all three IAT tasks (IAT 1, 493 ms in the congruent blocks vs. 553 ms in the incongruent blocks, *t*[23] = 2.99, adjusted *p* <.05; IAT 2, 498 vs. 548 ms, *t*[23] = 3.05, adjusted *p* <.05; IAT 3, 503 vs. 554 ms, *t*[23] = 3.31, adjusted *p* <.01). The overall mean RTs across the three tasks also showed a significant difference between the congruent and incongruent conditions (498 vs. 551 ms, *t*[23] = 4.48, adjusted *p* <.05).

In IAT 1, we confirmed the significant color-shape congruency effect observed in Experiment 1. Participants responded faster when circle-red and square-blue were assigned to the same response key (circle-red/square-blue vs. circle-blue/square-red condition; in Experiment 1: 502 vs. 544 ms; in Experiment 2: 493 vs. 533ms; in Makin and Wuerger [11]: 601 vs. 630ms). Combined with these results, we suggest that people tend to associate circles with red, squares with blue. In IAT 2, RTs in congruent conditions were significantly faster than were those in incongruent conditions, which indicated that a congruency effect for circle-red and triangle-yellow modulated RTs. In IAT 3, we observed a significant difference in RTs in congruent and incongruent conditions, such that participants responded faster in congruent conditions. This result indicates that color-shape associations between triangle and yellow and between square and blue influenced participants’ RTs. In all, across the three IATs, we observed that consistent associations of circle-red, square-blue, and triangle-yellow facilitated participants’ RTs for stimuli discrimination, which provides strong evidence that our participants encoded color-shape associations. Thus, we demonstrated that associations between colors and shapes could influence people’s stimulus discrimination and task performance at the level of response selection (i.e., circle-red, square-blue, triangle-yellow).

### Explicit questionnaire

Thirteen out of 24 of participants (54.17%) made color-shape combinations consistent with those of the present experimental sets (i.e., circle-red, square-blue, and triangle-yellow; Label 1 in Table 3). As only 24 participants were recruited in Experiment 2 and chosen times for circle-yellow, square-blue, and triangle-red associations were 0 (Label 5 in Table 3), we divided participants into two groups: Japanese color-shape association (Label 1 in Table 3; expected population rate = 16.7%) and the other associations (expected population rate = 83.3%). As a result of chi-square test, we found that Japanese color-shape association was chosen more than the other groups (*p* <.001). However, again this could be due to the IAT performed before the explicit matching questionnaire.

For participants who explicitly showed color-shape combinations consistent with our hypothesis, D scores compared with zero in IAT 1 and IAT 2 showed significant congruency effects while those in IAT 3 did not show the effects (IAT 1: *t*[12] = 4.73, adjusted *p* <.01, mean D score = 0.52; IAT 2: *t*[12] = 4.57, adjusted *p* <.01, mean D score = 0.45; IAT 3: *t*[12] = 2.36, adjusted *p* = .10, mean D score = 0.33). For participants who made unmatched explicit color-shape associations, all the IATs did not show significant association effects (IAT 1: *t*[10] = 0.90, adjusted *p* >.99, mean D score = 0.16; IAT 2: *t*[10] = 0.93, adjusted *p* >.99, mean D score = 0.16; IAT 3: *t*[10] = 2.75, adjusted *p* = .06, mean D score = 0.32). This may suggest that color-shape associations are demonstrated by both indirect behavioral performance and explicit matching tests; faster responses in the indirect behavioral method were caused by the same associations in the explicit matching task.

## General Discussion

In the present study, we used IATs to examine the associations between colors and shapes according to Kandinsky’s correspondence theory (circle-blue, square-red, triangle-yellow; Experiment 1) and the previous finding for color-shape associations among Japanese people (circle-red, square-blue, triangle-yellow; Experiment 2). The results of Experiment 1 showed little evidence for Kandinsky’s color-shape associations, which was consistent with the results that Makin and Wuerger [11] obtained with British participants. In Experiment 2, participants responded more rapidly when circle-red, square-blue, and triangle-yellow were mapped onto the same response key than when they were paired with different response keys. These results suggest robust effects for color-shape associations, in line with those observed in the previous Japanese sample [6]. Therefore, the present study confirmed the existence of associations between colors and shapes, which can be measured by the indirect behavioral methods.

To the best of our knowledge, this is the first study to demonstrate an association between colors and shapes using an indirect performance method. Studies using explicit matching tasks have reported different patterns of color-shape associations [3–8]. However, little has been demonstrated using indirect behavioral tasks. Previous studies using indirect performance methods applied Kandinsky’s correspondence theory and failed to provide clear evidence for color-shape associations [9–11]. Consistent with the results of our previous study using an explicit matching task (i.e., circle-red, square-blue, and triangle-yellow), the present study confirmed the existence of associations between colors and shapes by indirect behavioral methods.

In Experiment 1, we observed a congruency effect for color-shape associations in two IAT tasks. In IAT 1, pairing circle-red and square-blue with the same response key enhanced RTs. In IAT 3, triangle-yellow and square-red also showed a congruency effect on RT. In IAT 2, no color-shape association effect was observed (circle-blue and triangle-yellow). It seems contradictory that squares were paired with different colors in two IAT tasks (that is, blue and red, respectively). We suspect that this might be the due to the relative strength of the different color-shape associations (i.e., circle-red, triangle-yellow, square-blue). For example, in our previous study [6], we asked participants to choose the best-matched color for a shape from a 40 hue color wheel, twice. Results showed that 34% of participants chose red colors for a circle, 33% chose yellow colors for a triangle, and 19% chose blue colors for a square. Moreover, when comparing the first and second choices, the most consistent color choice was the selection of red for the circle, suggesting a stronger, more stable link for circle and red than for the other color-shape combinations. The square showed the least consistent color choice, being associated with blue, blue-green, and green, and triangle-yellow was the second most consistent association. Those results likely reflected to the relative strength of different color-shape associations. It may be that different strengths of color-shape associations led to the associations in IAT 1 (circle-red) and IAT 3 (triangle-yellow), as well as the null results of IAT 2 (circle-blue). Importantly, the varying strength of the color-shape associations might be influenced by cultural background to some extent. For example, strong association between circle and red among Japanese participants might be influenced by a salient cultural mediation factor: e.g., the Japanese flag has a red circle at the center of a white background. However, previous studies have also observed these associations in various other countries. For example, Albertazzi et al. observed circle-red, square-red, and triangle-yellow associations in Italian people [5]. Kharkhurin conducted a color-shape correspondence survey and found a square-blue association in participants from mixed cultural backgrounds [9]. Makin and Wuerger observed a marginal effect for square-red and triangle-yellow associations in British participants [11]. It would be interesting in a future study to investigate possible cultural differences in color-shape associations using indirect performance measures.

In the present study, the explicit questionnaire was conducted after the IATs. The choices might be influenced by the IAT tasks, because in the present experimental paradigms, the congruent color-shape associations appeared twice while the incongruent color-shape associations appeared only once. Nevertheless, the choices were probably consistent with the previous study using an explicit matching task [6]. From overall choices in Experiment 1 and 2 (N = 62), 34 participants (54.8%, chance level = 33.33%) chose associations including circle-red, 29 participants (46.77%) chose associations including square-blue, and 44 participants (70.96%) chose associations including triangle-yellow. In the previous study [6], Chen et al. instructed participant to choose the best matched color for a shape from 40 color circles (8 categorical colors; yellow, orange, red, purple, blue, blue-green, green, and yellow-green), and allowed them to choose the same color for various shapes. Then, Chen et al. found that about 34% of participants (chance level = 12.5%) matched circle with red colors, 19% of participants matched square with blue colors, and 33% of participants matched triangle with yellow colors. Although we could not statistically compare the results because of the different experimental paradigms, we may be able to mention that circle-red, square-blue, and triangle-yellow associations were selected more than chance level both in the present and previous studies [6]. Particular, participants who explicitly showed unmatched color-shape associations with Kandinsky’s theory in Experiment 1 responded faster in circle-red, square-red/blue, or triangle-yellow association. In addition, those who explicitly showed Japanese color-shape associations in Experiment 2 responded faster in circle-red, square-blue, or triangle-yellow association. These results indicate that faster responses in the indirect behavioral method are reflected by explicit color-shape associations which participants answered.

In summary, we observed evidence supporting our previous findings regarding color-shape associations in Japanese participants (i.e., circle-red, square-blue, and triangle-yellow) using the IAT method. We demonstrated that the robust color-shape associations seem to influence people’s RT for shape and color discriminations. These color-shape associations show little support for Kandinsky’s correspondence theory, but are fully in line with the color-shape associations we previously found among Japanese people.

## Supporting Information

S1 Table Individual information and task performance about D score and RT in each IAT session in Experiment 1.
(PDF)

S2 Table Individual information and task performance about D score and RT in each IAT session in Experiment 2.
(PDF)
