# Supplementary material for: Color-Shape Associations Revealed with Implicit Association Tests
Source: PLoS One. 2015 Jan 27;10(1):e0116954. doi: 10.1371/journal.pone.0116954 (PMC4308101; doi:10.1371/journal.pone.0116954)
Supplement: S1 Table — Individual information and task performance about D score and RT in each IAT session in Experiment 1. (PDF) [file pone.0116954.s001.pdf]

Table S1. Individual information and task performance about D score and RT in each IAT session in Experiment 1.

Participant information			Performances in IAT 1			Performances in IAT 2			Performances in IAT 3			All three IATs	
				RT (ms)			RT (ms)			RT (ms)			
ID	Age	Gender	D score	Congruent	Incongruent	D score	Congruent	Incongruent	D score	Congruent	Incongruent	Errors (%)	RT > 2000ms (%)
1	22	F	-0.07	451.22	453.46	0.89	381.64	487.57	-0.08	432.06	427.42	5.83	0.00
2	21	F	0.05	626.76	621.91	-0.26	550.00	520.02	-0.17	624.98	607.47	6.94	2.50
3	22	M	-0.18	514.07	497.19	0.44	491.04	567.80	1.00	491.15	692.11	1.94	0.28
4	20	M	0.73	447.26	564.53	0.01	559.08	589.75	0.17	647.00	685.02	9.17	1.11
5	21	F	-0.31	508.62	471.86	0.02	485.44	506.56	0.17	469.07	481.27	0.83	0.56
6	20	M	-0.41	531.91	481.60	-0.31	467.43	437.82	0.12	435.53	444.29	3.61	0.56
7	22	M	-0.24	509.91	493.70	-0.14	441.86	434.02	0.20	531.71	564.31	2.50	0.00
8	22	F	-0.58	464.49	404.52	-0.70	447.08	381.64	0.37	426.73	473.70	6.67	0.00
9	18	M	-0.54	628.96	545.48	0.93	496.73	731.63	0.57	544.37	674.18	1.94	1.94
10	20	M	-0.01	382.33	379.84	0.09	452.26	451.64	-0.54	411.61	375.52	3.61	0.28
11	24	M	-0.08	474.47	446.60	0.37	519.09	552.15	-0.12	481.98	478.94	3.89	0.00
12	19	M	0.58	391.48	464.31	0.62	446.92	540.43	0.44	385.72	433.20	4.17	0.56
13	23	M	-0.98	780.64	505.47	-0.69	494.13	436.70	-0.40	426.80	395.56	7.50	0.00
14	22	M	-0.95	660.70	473.05	0.42	512.65	598.15	0.69	503.76	594.53	3.61	5.00
15	22	M	-0.84	534.57	450.50	-0.67	434.97	389.14	-0.23	394.53	384.75	1.67	0.28
16	24	M	-0.79	465.28	391.77	-0.30	448.19	417.30	0.42	403.42	414.78	2.50	0.00
17	21	F	-0.14	454.29	447.30	-0.77	554.43	419.19	0.67	400.39	501.49	3.06	0.56
18	23	M	-0.29	698.02	617.21	-0.18	626.76	624.87	0.72	571.26	700.40	4.72	1.39
19	21	F	-0.48	609.09	529.70	-0.04	490.91	486.14	0.21	476.95	504.95	1.94	0.00
20	20	M	-0.14	441.73	422.67	0.15	514.76	528.41	-0.08	487.17	471.71	2.50	0.28
21	19	F	0.00	616.93	616.57	-0.18	555.51	504.33	-0.54	562.28	484.62	10.28	2.78
22	22	F	-0.93	796.68	522.68	0.15	481.04	513.68	0.52	492.46	565.75	0.56	0.28
23	23	M	-0.64	663.44	518.14	0.24	575.51	598.19	0.89	481.46	606.31	2.78	0.28
24	22	F	0.42	444.70	489.79	-0.30	497.98	477.55	0.62	465.47	592.96	4.17	0.83
25	20	M	0.10	559.75	563.19	0.12	568.20	588.93	-0.06	649.78	610.97	0.28	0.83
26	22	M	-0.07	674.72	660.65	0.10	635.11	669.16	0.64	530.76	689.96	0.83	0.83
27	20	M	-0.49	410.20	369.22	-0.15	399.50	391.57	0.29	409.31	440.42	7.78	0.00
28	24	M	-0.01	589.98	586.10	0.55	475.34	545.31	0.11	439.75	453.86	4.44	0.83
29	21	F	0.30	510.06	515.45	-0.10	431.59	425.73	0.09	466.46	466.07	5.83	0.56
30	20	M	-0.53	617.72	520.57	-0.02	582.14	580.02	0.69	506.84	645.04	0.83	1.11
31	22	M	-0.46	543.67	486.14	0.92	498.07	614.72	-0.18	545.59	519.35	0.00	0.00
32	22	M	-0.73	511.75	453.71	-0.26	510.82	461.39	0.60	465.14	549.62	2.78	0.28
33	24	M	-0.33	594.33	550.02	0.54	510.40	571.36	0.94	578.80	787.05	0.56	0.83
34	21	M	0.80	457.98	592.39	0.12	533.52	555.53	1.05	510.65	673.14	1.11	0.28
35	19	F	0.21	427.77	456.95	0.70	414.53	512.63	0.83	498.62	658.30	2.78	0.28
36	22	F	-0.58	580.33	519.74	-0.16	528.70	500.95	0.84	478.43	589.02	5.00	0.00
37	19	M	-1.25	906.02	468.66	0.43	595.78	645.20	1.37	467.20	1004.06	6.67	2.78
38	30	F	-0.11	929.04	902.54	-0.15	890.36	833.26	1.48	557.80	940.60	1.94	2.78

Note: F = Female; M = Male; RT = Response time in the correact trials after exclusion of trials whose RTs were longer than 2000ms.
