# Supplementary material for: Color-Shape Associations Revealed with Implicit Association Tests
Source: PLoS One. 2015 Jan 27;10(1):e0116954. doi: 10.1371/journal.pone.0116954 (PMC4308101; doi:10.1371/journal.pone.0116954)
Supplement: S2 Table — Individual information and task performance about D score and RT in each IAT session in Experiment 2. (PDF) [file pone.0116954.s002.pdf]

Table S2. Individual information and task performance about D score and RT in each IAT session in Experiment 2.

Participant information			Performances in IAT 1			Performances in IAT 2			Performances in IAT 3			All three IATs	
				RT (ms)			RT (ms)			RT (ms)			
ID	Age	Gender	D score	Congruent	Incongruent	D score	Congruent	Incongruent	D score	Congruent	Incongruent	Errors (%)	RT > 2000ms (%)
1	24	M	0.96	414.32	597.32	0.78	424.55	521.05	0.18	464.54	484.66	1.39	0.00
2	22	M	0.72	503.20	645.44	0.81	487.68	638.73	0.31	529.34	599.30	1.39	1.67
3	23	F	0.53	444.34	506.25	0.07	442.12	453.48	0.60	436.05	496.06	3.33	0.28
4	21	F	0.24	727.59	772.30	0.20	634.86	689.05	0.39	539.98	617.05	1.39	3.06
5	20	M	0.44	421.79	463.89	-0.41	480.47	439.57	0.67	440.31	522.46	2.22	0.00
6	20	M	-0.54	464.84	405.00	0.61	426.56	471.50	-0.34	481.24	427.88	9.44	1.67
7	20	F	0.34	411.33	459.45	0.90	373.02	526.02	-0.56	501.32	431.12	2.78	0.28
8	22	M	0.20	491.18	508.47	-0.49	591.94	479.56	-0.08	596.50	572.49	7.22	0.28
9	19	M	0.54	461.60	530.94	-0.41	521.42	474.84	-0.01	442.35	456.17	8.06	0.56
10	22	F	0.36	460.95	497.89	0.81	493.31	620.13	0.72	416.19	509.33	1.11	0.28
11	20	M	0.89	420.80	555.11	0.73	464.33	568.91	0.91	430.85	521.35	3.06	0.00
12	24	M	0.58	437.07	495.51	0.55	547.27	630.45	0.78	393.96	465.22	4.72	1.39
13	21	M	-0.12	600.55	579.50	0.64	508.67	603.06	0.51	633.65	734.44	5.28	1.94
14	20	M	0.91	414.72	500.61	0.30	412.57	432.32	0.91	429.19	533.23	3.33	0.00
15	21	M	0.39	577.15	694.85	0.70	543.57	712.00	0.82	534.37	809.42	2.50	5.83
16	21	F	0.44	408.57	465.65	-0.18	409.34	390.57	0.65	385.08	440.47	11.11	0.00
17	22	M	1.32	446.29	781.67	0.19	583.56	586.44	0.66	501.38	615.12	7.78	4.72
18	18	F	-1.04	587.64	407.95	0.73	499.67	621.24	0.05	562.31	559.26	5.00	0.28
19	20	M	-0.26	560.75	528.67	0.09	504.82	510.55	0.37	703.98	749.53	1.94	0.83
20	22	M	0.76	576.57	741.96	0.62	560.55	682.37	0.42	590.68	696.78	1.67	5.56
21	21	F	0.66	539.68	630.25	0.80	556.48	698.91	-0.34	627.85	562.73	1.11	1.39
22	20	M	0.08	497.50	503.71	-0.04	473.36	471.00	0.18	490.38	515.32	0.56	0.00
23	21	M	0.30	509.71	554.73	0.01	480.44	485.95	-0.52	510.95	459.50	1.67	0.56
24	20	F	-0.21	463.33	442.79	-0.50	550.46	461.04	0.59	434.11	520.22	5.28	0.28

Note: F = Female; M = Male; RT = Response time in the correact trials after exclusion of trials whose RTs were longer than 2000ms.
